# Bactericidal Activity of Lactic Acid against Clinical, Carbapenem-Hydrolyzing, Multi-Drug-Resistant *Klebsiella pneumoniae* Planktonic and Biofilm-Forming Cells

**DOI:** 10.3390/antibiotics8040181

**Published:** 2019-10-09

**Authors:** Taniya Bardhan, Madhurima Chakraborty, Bornali Bhattacharjee

**Affiliations:** National Institute of Biomedical Genomics, Kalyani, West Bengal 741251, India; tb1@nibmg.ac.in (T.B.); mc2@nibmg.ac.in (M.C.)

**Keywords:** *Klebsiella pneumoniae*, carbapenem-hydrolyzing, lactic acid, meropenem, planktonic, biofilm-forming, bactericidal

## Abstract

Carbapenem resistant *Klebsiella pneumoniae* has been highlighted to be a critical pathogen by the World Health Organization. The objectives of this study were to assess the efficacy of lactic acid (LA) against planktonic cells and biofilms formed by carbapenem-hydrolyzing *K. pneumoniae* isolates obtained from the nares of preterm neonates. Time-kill assays with graded percentages of (*v*/*v*) LA in water were initially carried out against planktonic cells of a meropenem (MRP)-resistant *K. pneumoniae* isolate, JNM11.C4. The efficacy parameters such as optimal incubation time and minimum inhibitory concentration were determined by comparing colony-forming unit counts (log_(10)_ CFU). Scanning electron microscopy was used to visualize cell damage. Likewise, JNM11.C4 biofilms were treated with graded series of (*v*/*v*) LA. Six carbapenem-hydrolyzing isolates were next used to validate the results. A reduction of 3.6 ± 0.6 log_(10)_ CFU/mL in JNM11.C4 planktonic cells and >3 ± 0.03log_(10)_ CFU/mL in biofilm-forming cells were observed using 0.225% and 2% LA, respectively, after three hours. Similar decreases in viable cell-counts were observed both in the case of planktonic (˃3.6 ± 0.3log_(10)_ CFU/mL) and biofilm-forming cells (3.8 ± 0.3log_(10)_ CFU/mL) across all the six clinical isolates. These results indicate that LA is an effective antimicrobial against planktonic carbapenem-hydrolyzing *K. pneumoniae* cells and biofilms.

## 1. Introduction

*Klebsiella pneumoniae,* a Gammaproteobacteria is a known commensal residing in the mucosal surfaces of the human body such as the gastrointestinal (GI) tract and the oropharynx [1]. However, it is also the leading cause of nosocomial infections associated with high mortality in the immunocompromised and nasal carriage of multi-drug-resistant (MDR) *K. pneumoniae* has also been reported among preterm neonates admitted to the sick neonatal care unit with respiratory distress [2,3]. The repertoire of diseases caused by *K. pneumoniae* in hospital wards is often associated with the prolonged use of infected medical equipment [4,5]. *K. pneumoniae* has extensive biofilm-forming ability; therefore, can form biofilms on respiratory support equipment and urinary catheters upon extended usage [6,7]. With the increase in use of antibiotics, the incidence of multi-drug resistance and extended spectrum β-lactamase (ESBL) carriage rates have also increased within this species, contributing to the inadequacy in treatment strategies and associated high mortality rates [8,9]. Hence, in 2017, carbapenem-resistant *K. pneumoniae* was declared a World Health Organization (WHO) priority level 1 critical pathogen. 

Lactic acid (LA), a bio-preservative in naturally fermented substances, has long been approved by the Federal Drug Administration (FDA) as a food antimicrobial and it also finds usage in the cosmetic industry [10,11,12]. Further, a number of recent reports have also highlighted the antimicrobial efficacy of LA alone or in combination against select gram-positive and gram-negative pathogens [13,14,15]. However, none of these reports have tested it is efficacy against *K. pneumoniae* cells or biofilms in general. Biofilms formed by bacterial communities are known for their impermeability attributed to the self-surrounding robust extracellular matrices made of eDNA, polysaccharides, and proteins [16]. Antimicrobials and disinfectants often prove to be ineffective against biofilms while being effective against planktonic bacterial cells because of the matrix [17].

Hence, the aim of this study was to gauge the efficacy of lactic acid, a known outer membrane-disintegrating agent and a weak antimicrobial [13], against both planktonic cells and biofilms formed by carbapenem-hydrolyzing clinical *K. pneumoniae* isolates.

## 2. Results

### 2.1. A Broad Range of Meropenem (MRP) Minimum Inhibitory Concentration (MIC) Values and Moderate Biofilm-Formation Was Determined across the K. pneumoniae Isolates

**Figure 1:**
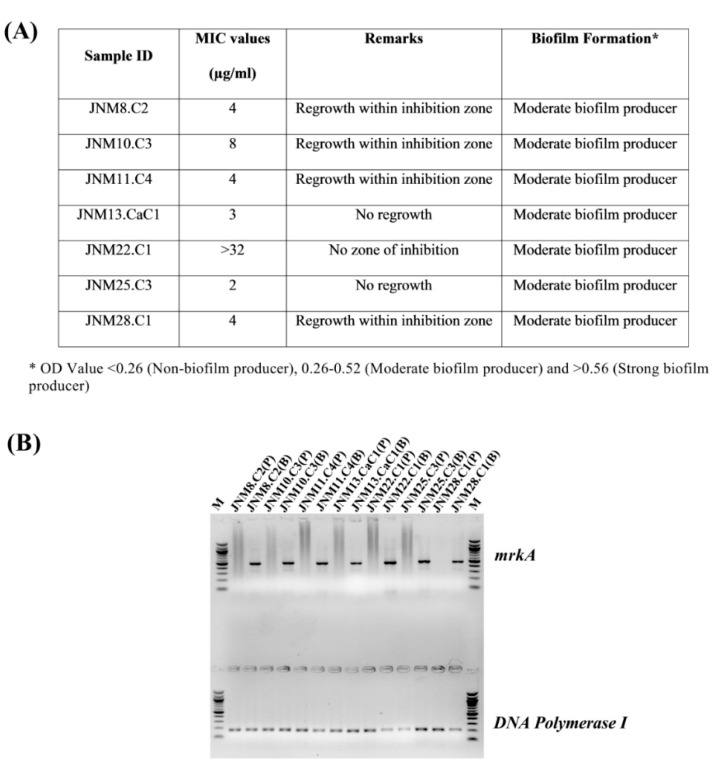
Characteristics of the K. pneumoniae clinical isolates. (**A**) Summary of the meropenem MIC values and biofilm forming abilities. (**B**) Validation of biofilm formation in 96-well static cultures after 24 hours using mRNA expression markers. The mrkA semi-quantitative RT-PCR products, 475 bp in size, were run on the wells above and the DNA polymerase I amplicons, 186 bp in size served as controls and were run on the lower lanes of a 1.5% agarose gel. M; 100 bp marker, P; planktonic cells, B; biofilm-forming sessile cells. All the bacterial cells recovered from biofilms across isolates expressed the mrkA gene while none of the planktonic cells had any detectable expression of the same.

The MRP MIC values ranged between 2 to >32 µg/mL and JNM22.C1 had the highest MIC value of >32 µg/mL. Some of the isolates, namely JNM8.C2, JNM10.C3, JNM11.C4, JNM13.CaC1, and JNM28.C1, were found to exhibit heteroresistance. All the isolates were also found to be moderate biofilm producers (Figure 1A). All the sessile cells across isolates had *mrkA* gene expression validating the formation of biofilms (Figure 1B). 

### 2.2. Bactericidal Activity of LA against Planktonic K. pneumoniae, JNM11.C4 Cells In Vitro

**Figure 2:**
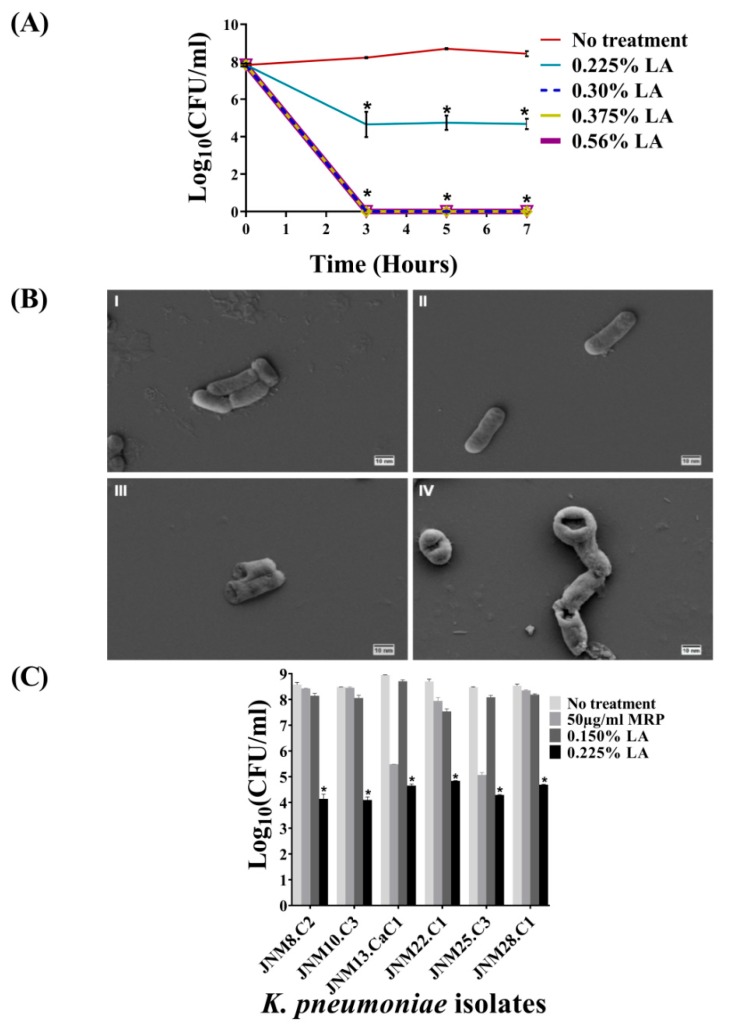
Lactic acid (LA) kills carbapenem-hydrolyzing, third generation cephalosporin resistant K. pneumoniae isolates. (**A**) Mid-logrithmic JNM11.C4 cells were exposed to different percentages (*v*/*v*) of lactic acid (LA) for 3, 5 and 7 hours. Experiments were done in duplicate and error bars represent mean ± standard deviation. After 3, 5 and 7 hours the average log_(10)_ CFU/mL values were >8. Treatment with 0.225% (LA) resulted in >3log_(10)_ CFU/mL reduction across time points and no visible growth was observed at 0.30% (*v*/*v*) LA or above. The differences in log_(10)_ CFU/mL values between untreated and treated JNM11.C4 cells were statistically significant after multiple testing correction. (**B**) Scanning electron micrographs of JNM11.C4 cells at 20,000× magnification (I) untreated; (II) Meropenem (MRP) treated; (III) 0.150% LA treated; (IV) 0.225% LA treated for three hours. LA treatment resulted in cell membrane damage. **(C)** Six additional carbapenem-hydrolyzing isolates with different MRP MIC values were tested for bactericidal efficacy using 0.150% (*v*/*v*) and 0.225% (*v*/*v*) LA for 3 hours. Treatment with 0.225% (*v*/*v*) LA resulted in >3.5log_(10)_ CFU/ml reduction across isolates and these differences were also found to be statistically significant. Reduction values of ≥3log_(10)_ CFU/ml upon treatment with *p*-values of <0.05 were considered to be bactericidal (denoted by astericks).

The results of the bactericidal activity of graded series of LA (*v*/*v*) on planktonic JNM11.C4 cells across three incubation time points, namely, 3, 5, and 7 h, are shown in Figure 2A. For JNM11.C4 isolate the MIC value was determined to be 0.3% with no visible growth and an average reduction of 3.8 ± 0.37log_(10)_ CFU/mL was recorded with 0.225% LA across time-points and this was considered to be the MIC_50_ value. The decreases were statistically significant (Supplementary Table S1). Given that 3 h incubation was as efficacious as 5 or 7 h, subsequent killing assays were carried out for 3 h.

### 2.3. Treatment with LA Caused Membrane Damage

Untreated and the cells treated with MRP for 3 h were visualized using SEM and found to retain smooth membrane surfaces and cell shape. By contrast, both 0.150% and 0.225% LA-treated JNM11.C4 cells exhibited significant morphological changes, membrane distortion, and rupture (Figure 2B, Supplementary Figure S1).

### 2.4. Bactericidal Activity of LA across K. pneumoniae Isolates

In order to validate the efficacy of 0.225% LA in killing carbapenem-hydrolyzing MDR planktonic *K. pneumoniae* cells in vitro, six additional clinical isolates (Supplementary Table S1) were tested. Across isolates, reduction of 3.8 to 4.4 ± 0.3log_(10)_ CFU/mL were recorded confirming the average MIC_50_ value of LA to be 0.225%, and these differences were statistically significant (Figure 2C, Supplementary Table S2). Among the intermediately susceptible isolates, with the exception of JNM8.C2, a reduction of >3log_(10)_ CFU/mL was also observed after MRP treatment.

### 2.5. Bactericidal Activity of LA against Sessile K. pneumoniae Cells Forming Biofilms

**Figure 3:**
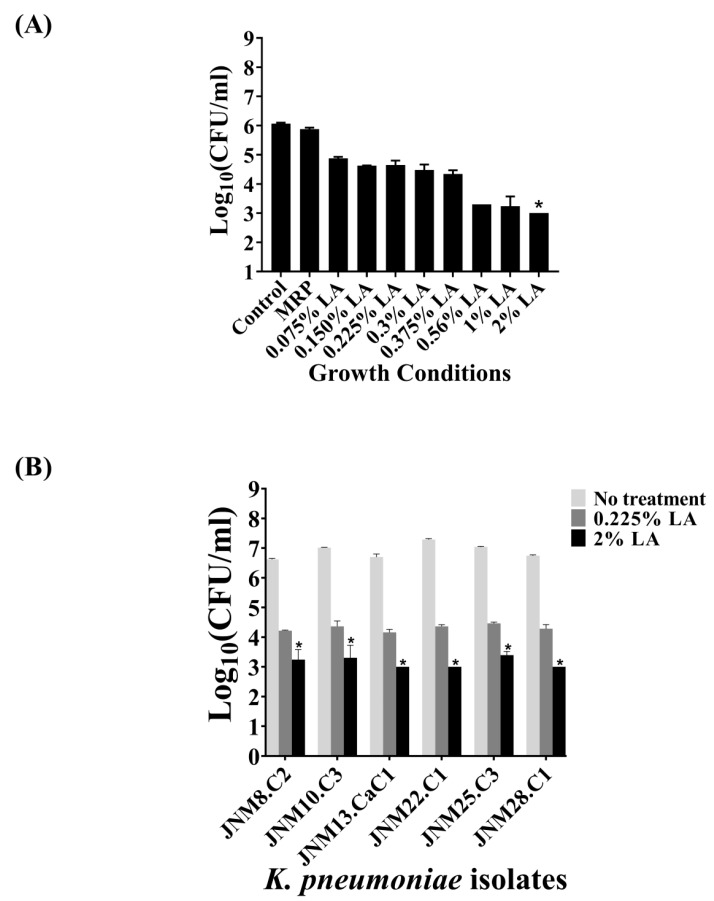
Lactic acid penetrates biofilms formed by carbapenem-hydrolyzing, third generation cephalosporin resistant *K. pneumoniae* and kills sessile cells. (A) The bactericidal efficacy on biofilm-forming JNM11.C4 cells was tested using 0.075%–2% (*v*/*v*) LA. An average reduction of 3 log_(10)_ CFU/mL was observed at 2% (*v*/*v*) LA. (B) The efficacy of 0.225% and 2% (*v*/*v*) LA were tested in rest of the *K. pneumoniae* isolates. Reduction values of ≥ 3log_(10)_ CFU/ml upon treatment with *p*-values of <0.05 were considered to be bactericidal (denoted by astericks).

The bactericidal efficacy of LA against JNM11.C4 sessile, biofilm-forming cells was next tested using a graded series of LA (*v*/*v*), namely 0.075%, 0.150%, 0.225%, 0.3%, 0.375%, 0.56%, 1%, and 2% for 3 h. The series was comprised of the same LA percentages used in treatment of the planktonic cells along with lower and higher percentages to accommodate the emergent cellular properties associated with biofilm formation. The highest reduction was observed with 2% LA with a statistically significant average reduction of 3log_(10)_ CFU/mL (adjusted *p*-value = 0.021). However, 0.225% LA that had caused a reduction of >3log_(10)_ CFU/mL in planktonic JNM11.C4 cells was not effective against the sessile cells with a negligent decrease of 1.4 ± 0.18 log_(10)_ CFU/mL. The results are shown in Figure 3A and the adjusted *p*-values are tabulated in Supplementary Table S3. The bactericidal efficacy of both 0.225% and the MIC_50_ value of 2% (*v*/*v*) LA were tested across isolates. The reduction of log_(10)_ CFU/mL ranged from 3.4 to 4.0 ± 0.3log_(10)_ CFU/mL when the sessile cells were treated with 2% (*v*/*v*) LA across isolates. The reduction in log_(10)_ CFU/mL were also found to be statistically significant (Figure 3B, Supplementary Table S3).

## 3. Discussion

The goal of this study was to examine the use of LA, a generally recognized safe (GRAS) compound approved by the FDA [10], as an antimicrobial agent against carbapenem-hydrolyzing, MDR *K. pneumoniae* isolates. In practice, the last-resort treatment against carbapenem-resistant *K. pneumoniae* infections includes monotherapy or combined administration of tigecycline or polymyxins. However, there is growing evidence of the spread of tigecycline resistance [18]. Similarly, the unregulated use of polymyxins such as colistin in the poultry and meat industry as growth promoters has also resulted in dissemination of colistin resistance across species [19]. Hence, to prevent the spread of such pathogens within hospitals, disinfection of equipment, catheters, and tubes need to be stringent. The WHO disinfection protocol prescribes soaking of hospital instruments in formaldehyde, glutaraldehyde, or chlorhexidine and require subsequent washing-off of these chemicals [20]. Both formaldehyde and glutaraldehyde are categorized as hazardous chemicals while chlorhexidine is a contact allergen [21]. All of these chemicals require careful handling and storage by health professionals. Hence, efficacious antimicrobials and safe disinfectants are the need of the hour to prevent the spread of antimicrobial resistance.

Earlier reports have highlighted the efficacy of different doses of LA against different genera of the class Gammaproteobacteria such as *Escherichia coli* and *Salmonella* spp. [10,15]. However, there are no reports to confirm the efficacy of LA treatment against *K. pneumoniae* or biofilms in general. Hence, this study was undertaken to deduce the efficacy of LA as an antimicrobial against carbapenem-hydrolyzing MDR *K. pneumoniae*. The percentages of LA used included those that had been reported to be efficacious against other members of the class Gammaproteobacteria, as well as higher percentages to confirm the MIC values for both planktonic and sessile cells. The results presented in this report support the bactericidal efficacy of LA against both planktonic as well as biofilm-forming carbapenem-hydrolyzing MDR *K. pneumoniae* cells in vitro. In summary, given the short exposure time of 3 h and the low percentage of LA (0.225% and 2%) required to achieve both planktonic and sessile cell death, the findings highlight the potential of LA as a cost-effective antimicrobial and disinfectant for efficacious removal of biofilms.

## 4. Materials and Methods

### 4.1. Determination of Minimum Inhibitory Concentration (MIC) Values of Meropenem (MRP)

Seven carbapenem-hydrolyzing *K. pneumoniae* clinical isolates, JNM8.C2, JNM10.C3, JNM11.C4, JNM13.CaC1, JNM22.C1, JNM25.C3, and JNM28.C1, were included in this study [16]. The meropenem (MRP) minimum inhibitory concentrations (MIC) were determined using MIC evaluator strips ranging from 0.002 to 32 µg/mL (Himedia Labs, Mumbai, India). Experiments were carried out according to Clinical and Laboratory Standards Institute guidelines [22]. The hospital ethical committee reference number is F-24/Pr/COMJNMH/IEC/16/536.

### 4.2. Analyses of Biofilm Formation

The biofilm-forming ability of the isolates were also quantitated as previously described [23]. Briefly, 96-well polystyrene, flat-bottom microtiter plates (Tarsons Products Pvt. Ltd., Kolkata, India) were filled with 180 μL of tryptic soy broth (TSB; Himedia Labs). Then, 20 μL of bacterial cells grown to a Macfarland score of 0.5 in brain heart infusion broth (BHI; Himedia Labs) were added and incubated at 37 °C for 24 h without shaking for biofilm formation. Each isolate was tested in triplicate and sterile TSB was used as negative control. After 24 h, crystal violet (Himedia Labs) binding assays were carried out.

To further validate the presence of biofilm-forming cells, *mrkA* gene expression was used as a marker. Bacterial cells were grown in 96-well microtiter plates as described. Planktonic cells in the broth were first collected. The wells were then washed once with PBS and the biofilm-forming cells were scraped off the bottom surface of the wells and collected in 200 µL PBS. Total cellular RNA was isolated using the Trizol reagent (Invitrogen, California, USA) according to manufacturer’s instructions. For this experiment, each isolate was grown in five independent wells and the cells were pooled together for RNA isolation. First strand cDNA synthesis was carried out using 500 ng total RNA, 500 µM of each deoxynucleoside triphosphate (dNTP; New England Biolabs, Ipswich, MA, USA), 200 ng random hexamers (Thermo Fisher Scientific, Waltham, MA, USA), and 3.75 U WarmStart^®^ RTx Reverse Transcriptase (New England Biolabs), followed by semi-quantitative reverse transcriptase PCR (RT-PCR), as described previously and *DNA Polymerase I* gene expression was used as an internal control [3,24].

### 4.3. Time Course LA MIC Determination Assays on Planktonic Cells

In-vitro broth dilution assays were initially carried out in duplicate using ~10^8^ JNM11.C4 planktonic cells grown in Muller-Hinton broth (MHB; Himedia Labs). In a 2015 report, treatment for an hour with 0.25% (*v*/*v*) LA was found to be effective in complete killing of planktonic *E. coli* cells [15]. By contrast, a 2017 report defined 0.2% (*v*/*v*) LA as the MIC for planktonic *E. coli* cells after 24 h of incubation [10]. Hence, the bacterial cells were treated with 0.225% (average of 0.2% and 0.25%), 0.3%, 0.375%, and 0.56% (*v*/*v*) LA (Himedia Labs) in a final volume of 200 µl for 3, 5, and 7 h, and colony-forming units (CFU) were calculated at each time-point to determine the bactericidal efficacy. The definition of bactericidal activity was set at ≥3 log_(10)_ CFU/mL reduction in viable cell counts in comparison to the original inoculum [25]. A constant value of one was added to all the CFU counts before log transformation to avoid undefined values.

### 4.4. Scanning Electron Microscopy (SEM)

To visualize bacterial membrane damage following treatment with LA, SEM was carried out as previously described [23]. JNM11.C4 cells were treated with 0.150% and 0.225% LA in triplicate, as described above for 3 h, while untreated and cells treated with 50 μg/mL MRP (Pharma Impex Laboratories Pvt. Ltd, Kolkata, India; a kind gift from Dr. Manjari Basu, COMJNMH, West Bengal, India) served as controls. The lower percentage of 0.150% (*v*/*v*) LA was used to ensure the presence of adequate number of cells for visualization in the presence of high rates of bacteriolysis. After treatment, cells were centrifuged at 6000 rpm for 5 min and washed thrice with PBS. Thereafter, bacterial cells were fixed in 2.5% (*v*/*v*) glutaraldehyde–PBS at 4 °C for 3 h, washed with PBS twice, and dehydrated using 500 μL graded ethanol series (30%, 50%, 60%, 80%, and 100%) for 15 min in each dilution. Ten microliter aliquots were loaded onto coverslips, desiccated, and visualized using a scanning electron microscope (SUPRA 55-VP; Ziess, Germany).

### 4.5. Validation of Bactericidal Activity

The bactericidal activity of LA and the effective percentages determined against planktonic and sessile JNM11.C4 cells were validated as described above on JNM8.C2, JNM10.C3, JNM13.CaC1, JNM22.C1, JNM25.C3, and JNM28.C1 isolates. Briefly, mid-logarithmic cells were grown in the presence of 0.150%, 0.225% LA and 50 µg/mL MRP in duplicate for 3 h and CFU counts were ascertained.

### 4.6. LA MIC Determination Assays on Biofilm-Forming Cells and Validation

The adherent JNM11.C4 biofilm-forming cells were treated with 200 μL of graded LA series (0.075%, 0.150%, 0.225%, 0.3%, 0.375%, 0.56%, 1%, and 2%) in duplicate for 3 h at 37 °C and a shaking speed of 200 rpm. The wells were washed once after treatment with PBS and the remaining adherent cells were resuspended in PBS. Cells treated with water and 50 μg/mL MRP served as controls. The CFU counts of the untreated wells were compared to the LA-treated wells to quantitate efficacy. The MIC_50_ determined was tested across isolates to validate the bactericidal efficiency.

### 4.7. Statistical Tests

One-way analyses of variance (ANOVAs) with Tukey’s multiple comparisons tests were performed using GraphPad Prism version 7.04 (GraphPad Software, La Jolla, California, USA) to identify significant differences among experimental conditions tested (*p*-value < 0.05).

## 5. Conclusions

Taken together, the experimental data presented here highlight the bactericidal efficacy of LA against MDR, carbapenem-hydrolyzing *K. pneumoniae* clinical isolates. LA was found to be efficacious against both planktonic and sessile biofilm-forming bacterial cells. However, in the MIC determination assays it was observed that the effective percentage of LA against planktonic cells was about 10-fold lower than that needed to kill sessile cells upon biofilm formation. 

## Supplementary Materials

The following are available online at https://www.mdpi.com/2079-6382/8/4/181/s1, Supplementary Figure S1: Scanning electron micrographs of JNM11.C4 cells at 10,000× magnification; Supplementary Table S1: Time-kill assay analyses results of the *K. pneumoniae* isolate, JNM11.C4 after multiple testing correction; Supplementary Table S2: The summary of the efficacy of LA against planktonic carbapenem-hydrolyzing *K. pneumoniae* cells across isolates, as determined using log_(10)_ CFU differences, and the *p*-values after multiple testing corrections for the relevant comparisons are tabulated here; Supplementary Table S3: Bactericidal efficacy results of different percentages of LA on JNM11.C4 isolate after biofilm formation. The tabulated *p*-values are those of relevant comparisons; Supplementary Table S4: The summary of the efficacy of lactic acid against sessile, biofilm-forming cells across isolates as determined using log_(10)_ CFU differences, and the *p*-values after multiple testing corrections for the relevant comparisons are tabulated here.
